# Gene and isoform expression signatures associated with tumor stage in kidney renal clear cell carcinoma

**DOI:** 10.1186/1752-0509-7-S5-S7

**Published:** 2013-12-09

**Authors:** Qi Liu, Shilin Zhao, Pei-Fang Su, Shyr Yu

**Affiliations:** 1 Center for Quantitative Sciences, Vanderbilt University School of Medicine, Nashville, TN 37232, USA; 2 Department of Biomedical Informatics, Vanderbilt University School of Medicine, Nashville, TN 37232, USA; 3 Department of Statistics, National Cheng Kung University, Taiwan; 4 Department of Cancer Biology, Vanderbilt University School of Medicine, Nashville, TN 37232, USA; 5 Department of Biostatistics, Vanderbilt University School of Medicine, Nashville, TN 37232, USA

## Abstract

**Background:**

Identification of expression alternations between early and late stage cancers is helpful for understanding cancer development and progression. Much research has been done focusing on stage-dependent gene expression profiles. In contrast, relatively fewer studies on isoform expression profiles have been performed due to the difficulty of quantification and noisy splicing. Here we conducted both gene- and isoform-level analysis on RNA-seq data of 234 stage I and 81 stage IV kidney renal clear cell carcinoma patients, aiming to uncover the stage-dependent expression signatures and investigate the advantage of isoform expression profiling for identifying advanced stage cancers and predicting clinical outcome.

**Results:**

Both gene and isoform expression signatures are useful for distinguishing cancer stages. They provide common and unique information associated with cancer progression and metastasis. Combining gene and isoform signatures even improves the classification performance and reveals additional important biological processes, such as angiogenesis and TGF−beta signaling pathway. Moreover, expression abundance of a number of genes and isoforms is predictive of the risk of cancer death in an independent dataset, such as gene and isoform expression of ITPKA, the expression of a functional important isoform of UPS19.

**Conclusion:**

Isoform expression profiling provides unique and important information which cannot be detected by gene expression profiles. Combining gene and isoform expression signatures helps to identify advanced stage cancers, predict clinical outcome, and present a comprehensive view of cancer development and progression.

## Background

Stepwise progression of cancer malignancy has been clinically well defined [1]. In the early stage, the cancer cells, confined to a very limited area, are not invasive and metastatic, whereas in the late stage, the cells, spreading to distant sites in the body, are highly invasive and metastatic. Comparative analysis of genetic, epigenetic, and expression alterations between early and late stage cancers can help to understand cancer progression and metastasis mechanisms and predict the clinical aggressiveness of cancer [1]. Many studies have been extensively performed on various types of human cancers [2-22]. For example, molecular mutations were reported to be accumulated in a fashion that paralleled the clinical progression of colorectal cancer [5,7,10]. Changes in DNA methylation were also found to be cumulative with disease progression in ovarian cancer, gastric cancer and prostate cancer [3,8,11]. Stage-dependent mRNA and microRNA expressions were identified in neuroblastoma, colon cancer, bladder cancer and gastric cancer [2,4,6,9]. Based on these discovered genetic, epigenetic, and expression alternations, models of tumor progression have been constructed, and the process of tumor progression and metastasis has been studied.

In addition to genetic, epigenetic, and expression alternations, post-transcriptional deregulation also plays an important role in cancer progression [17-23]. For example, alternative splicing of FGFR1 was found to be associated with tumor stage and grade; isoform switch of FGFR1 may result in a proliferative advantage that plays a key role during bladder tumor progression [18]. Alternative splicing leads to expression changes of specific isoforms, possibly without overall mRNA expression alternations. Isoform expression alternations, however, have not been widely studied partly due to the difficulty of isoform expression quantification. Recently, RNA-seq has been increasingly used to discover and profile the whole transcriptome [24]. The digital nature of RNA-seq technology coupled with powerful bioinformatics methods including Alexa-seq [25], IsoEM [26], Multisplice [27], MISO [28], Cufflinks [29,30], iReckon [31] and RSEM [32,33], which aim to quantify isoform expression accurately, provides the opportunity of systematically studying expression alternations at isoform level. However, due to the complexity of transcriptome and read assignment uncertainty, calculating isoform abundance from incomplete and noisy RNA-seq data is still challenging [34]. The advantage of using isoform expression profiles to identify advanced stage cancers and predict clinically aggressive cancers remains unclear.

In this study, we performed a comprehensive analysis on RNA-seq data of 234 stage I and 81 stage IV kidney renal clear cell carcinoma (KIRC) patients. We identified stage-dependent gene and isoform expression signatures and quantitatively compared these two kinds of signatures in terms of cancer stage classification, biological relevance with cancer progression and metastasis, and independent clinical outcome prediction. We found that isoform expression profiling provided unique and important information that could not be detected at the gene level. Combining isoform and gene signatures improved classification performance and presented a comprehensive view of cancer progression. Further examination of these signatures discovered well known and less studied gene and isoform candidates to predict clinically aggressive cancers.

## Methods

### RNA-seq data analysis of KIRC

Clinical information and expression quantification results of RNA-seq data for kidney renal clear cell carcinoma patients were downloaded from the website of Broad Institute's Genome Data Analysis Center (https://confluence.broadinstitute.org/display/GDAC/Home, 2013_02_03 stddata Run). In total, there are 480 cancer samples with RNA-seq data, including 234 stage I, 48 stage II, 117 stage III and 81 stage IV patients (Table 1). RSEM is used to estimate gene and isoform expression abundance, which is the estimated fraction of transcripts made up by a given isoform and gene [32,33]. Isoforms with expression larger than 0.001 TPM (transcript per million) in at least half of the stage I or stage IV samples were kept. Limma [35] was applied to identify differentially expressed genes and isoforms between 234 stage I and 81 stage IV patients using the criteria: (1) fold change (FC) ≥ 2 and (2) FDR ≤ 0.001(Benjamini and Hochberg's multiple-test adjustment). When significant changes were detected at both gene and isoform levels, only gene signatures were selected for further analysis.

**Table 1:** Characteristics of patients with RNA-seq data for kidney renal clear cell carcinoma

	Stage I (n = 234)	Stage II (n = 48)	Stage III (n = 117)	Stage IV (n = 81)
Age, years, mean ± SD	59.9 ± 12.8	58.4 ± 12.0	62.9 ± 12.1	60.8 ± 9.9
Gender, Male, n (%)	145 (62.0%)	36 (75.0%)	76 (65.0%)	56 (69.1%)
Median follow-up, month (minimum - maximum)	37.8 (0.1-112.6)	47.7 (0.1-94.3)	29.5 (0.1-96.0)	18.9 (0.1-87.0)
No. of deaths (%)	38 (16.2%)	8 (16.7%)	45 (38.5%)	64 (79.0%)

### Classification of cancer stages

Consensus clustering [36] was used to evaluate the effectiveness of gene and isoform signatures for separating early and late stage cancers. Consensus clustering is a resampling-based method to represent the consensus across multiple runs of a clustering algorithm. Given a data set of patients with a certain number of signatures, we resampled the data, partitioned the resampled data into two clusters, and calculated the classification score for each resampled dataset based on the agreement of the clusters with known stages. We defined the classification stability score (SS) as a properly normalized sum of the classification scores of all the resampled datasets (Eq.1). In the equation, the consensus matrix M(i,j) is the portion of the resampled dataset {D^(h) ^: h = 1,2,...,H} in which two patients i and j are clustered together, ${\text{s}}_{\text{i}}$ and ${\text{s}}_{\text{j}}$ are the known stages of patients i and j, and ES is the expected stability score of the perfect clustering where the entry in consensus matrix M equals 1 for patient pairs with the same stage and the entry equals 0 for patient pairs with different stages. We have 234 stage I and 81 stage IV patients, thus the expected score of the perfect clustering is 30501. The stability score estimates how sensitive the clustering results are to patient variability and indicates the classification performance to unknown samples. Here we used ConsensusClusterPlus package [37] to subsample signatures and patients 500 times, whereby a subset of gene/isoform signatures and patients (80%) was sampled without replacement from the original dataset. We implemented both hierarchical and kmeans clustering algorithms based on spearman correlation and the stability score of each algorithm was reported separately.

$$M\left(i,j\right)=\frac{\sum_{h}M^{\left(h\right)}\left(i,j\right)}{\sum_{h}I^{\left(h\right)}\left(i,j\right)}$$

(1) $$\text{SS}=\frac{\sum_{\text{i},\text{j},\text{i}<j}{\;\text{A}}_{\text{ij}}\text{M}\left(\text{i},\text{j}\right)}{\text{ES}}\;\;\;\;\;\;\;\left\{\begin{array}{c} {\text{A}}_{\text{ij}}=1,if\;{\text{s}}_{\text{i}}={\text{s}}_{\text{j}}\\ {\text{A}}_{\text{ij}}=-1,if\;{\text{s}}_{\text{i}}\neq {\text{s}}_{\text{j}} \end{array}\right)$$

$$ES=\underset{i,j,i<j}{\sum }A_{ij}M\left(i,j\right)\left\{\begin{array}{c} A_{ij}=1\;and\;M\left(i,j\right)=1,if\;s_{i}=s_{j}\\ A_{ij}=-1\;and\;M\left(i,j\right)=0,if\;s_{i}\neq s_{j} \end{array}\right)$$

### Function enrichment

Isoform names were converted into gene symbols by UCSC Genome Table Browser (http://genome.ucsc.edu/cgi-bin/hgTables). Functional enrichment analysis on the list of gene and isoform signatures was implemented in GO biological process as well as KEGG pathways by WebGestalt [38] (http://bioinfo.vanderbilt.edu/webgestalt/). Enrichment p-values were generated by a hypergeometric test and adjusted by Benjamini and Hochberg's multiple-test. Functional categories with FDR≤0.05 were selected.

### Survival analysis

165 stage II and stage III patients were used as an independent dataset to evaluate whether gene and isoform expression signatures were predictive of increased risk of cancer death by a Cox proportional hazard (PH) model. The patients were divided into two groups according to the median expression value of a given gene and isoform. Survival analysis was performed between higher- and lower-than-median groups. Genes and isoforms with FDR≤0.05 were considered to be significantly associated with clinical outcome. A multivariate model adjusting for age and gender of patients was also performed for selected genes and isoforms.

## Results

### Isoform profiles provide additional information

We estimated the alternative splicing activity and found that about 70% of multi-exon genes expressed two or more isoforms in each sample. This is consistent with the estimate by Griffith et al.[25], which reported 68% of multi-exon genes showed evidence for expression of multiple isoforms. We considered the isoform with the highest abundance as the "major" isoform and calculated the relative abundance ratio of the "major" isoform to the corresponding gene. For all genes, about 62% of the major isoforms had a ratio greater than 0.8 (Additional File 1A, the mean of ratio was 0.82, Median = 0.93, SD = 0.21). For genes with multiple isoforms, about 40% of major isoforms had a ratio greater than 0.8 (Additional File 1B, the mean of ratio was 0.71, Median = 0.72; SD = 0.20). These results indicate that one isoform is more highly expressed than the others for most genes.

**Figure 1:**
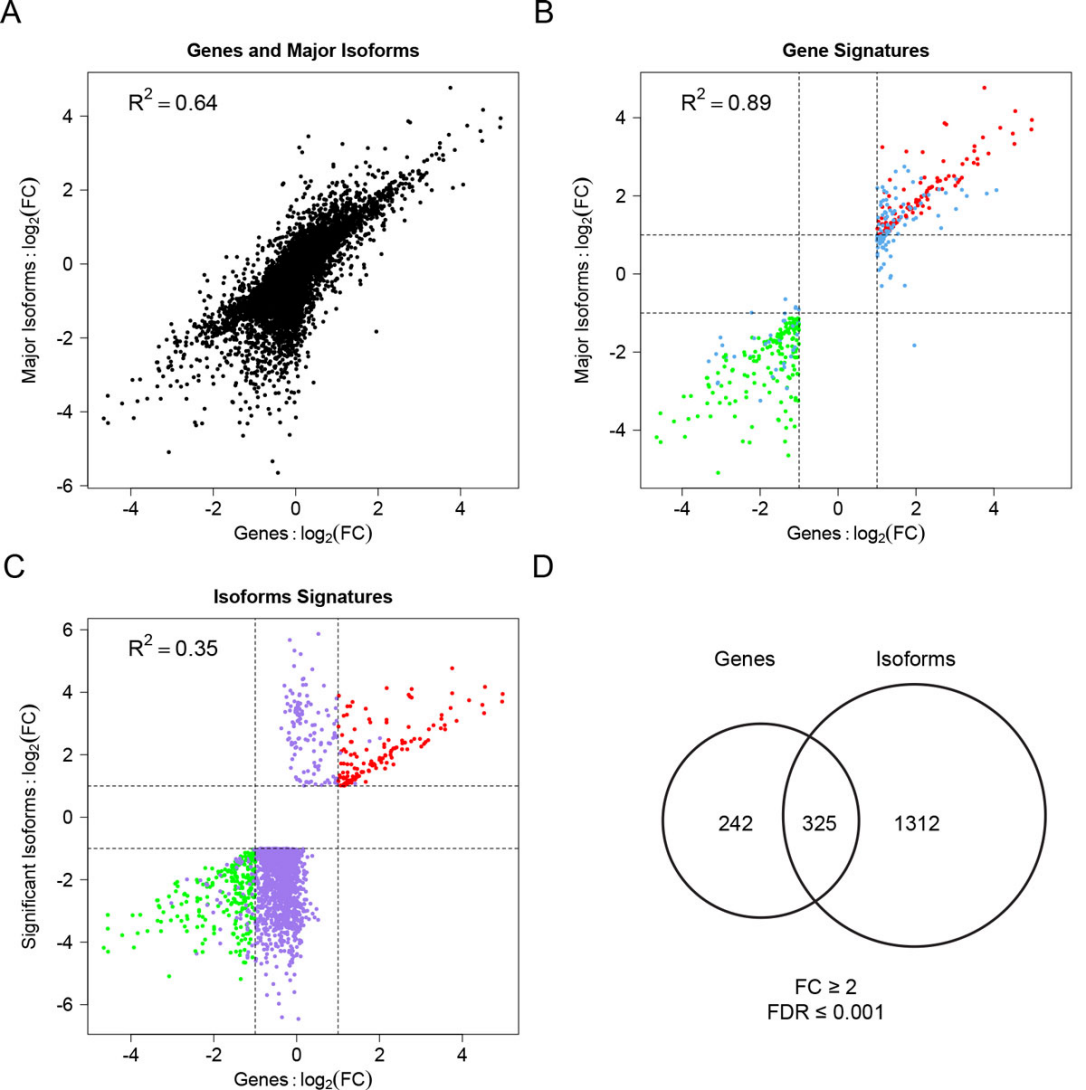
**Correlation of gene and isoform profiles in terms of log_2 _fold changes (FCs)**. **(A) **Correlation of genes and major isoforms. **(B) **Correlation of differentially expressed genes using the criteria of FC ≥ 2 and FDR ≤ 0.001 and their corresponding major isoforms. Red or green points depict both significant gene and major isoform up-regulation or down-regulation, respectively. Blue points denote only significant gene expression changes without major isoform expression changes. **(C) **Correlation of differentially expressed isoforms using the criteria of FC ≥ 2 and FDR ≤ 0.001 and their corresponding genes. Red or green points depict both significant gene and isoform up-regulation or down-regulation, respectively. Purple points denote only significant isoform expression changes without gene level changes. **(D) **Venn diagram showing the number of genes with significant overall mRNA expression changes and genes with significant isoform expression changes.

To compare the capacities of gene and isoform expression profiles to detect alternations, we calculated the fold change-based correlation between genes and their major isoforms. The correlation was high for all genes (Figure 1A, R^2 ^= 0.64, p<2.2e-16) and even higher if only differentially expressed genes (FC≥2 & FDR≤0.001) were considered (Figure 1B, R^2 ^= 0.89, p<2.2e-16), suggesting genes and their major isoforms are quite consistent in capturing expression changes. In contrast, the correlation of differentially expressed isoforms (FC≥2 & FDR≤0.001) and their corresponding genes was lower (Figure 1C, R^2 ^= 0.35, p<2.2e-16), which suggests isoform expression profiling provides additional information that cannot be detected at the gene level. This is possibly due to two reasons. One reason may be that isoform switching induces differential splice variants without gene-level expression changes; the other reason is that, with only one isoform altered, the addition of other isoforms to the total gene expression level simply obscures gene-level expression change.

In total, 567 genes showed significant expression changes between stage I and stage IV patients (FC≥2 & FDR≤0.001, Additional File 2). Interestingly, more genes (1637 vs. 567) were detected significant at the isoform level than the gene level (Additional File 3, Figure 1D). Among the 567 gene signatures, 325 genes (57%) had at least one isoform with significant expression change (Figure 1D). In contrast, only 20% of genes with significantly changed isoforms could be detected at the gene level. The remaining 80% of the genes with significant isoforms did not show significant changes at the gene level, which represents the unique information provided by isoform expression profiles.

For most genes with significantly changed isoforms, only one isoform was altered between early and late stage cancers. Notably, there were only 17 genes with two or more isoforms showing opposite expression changes, leading to no expression changes at the gene level. In these cases, isoform switching mainly contributes to isoform expression alternations. Among the 17 genes, half of them have been reported to be associated with cell proliferation or cancer progression (Additional File 4).

### Combining gene and isoform signatures improves cancer stages classification

**Figure 2:**
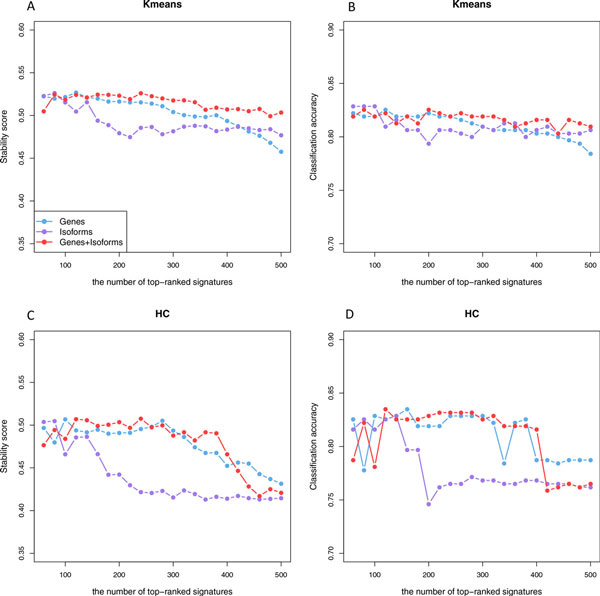
**The performance of gene and isoform signatures in separating cancer stages**. **(A) **The classification stability score obtained by gene, isoform and combined signatures with k-means clustering algorithms. **(B) **The classification accuracy obtained by gene, isoform and combined signatures with k-means clustering algorithms. **(C) **The classification stability score obtained by gene, isoform and combined signatures with hierarchical clustering algorithms. **(D) **The classification accuracy obtained by gene, isoform and combined signatures with hierarchical clustering algorithms.

Having identified stage-dependent gene and isoform expression signatures, one of the important questions is to assess the power of these signatures to classify unknown samples, which is essential for early cancer diagnosis. We applied consensus clustering [36], a resampling-based method to estimate classification stability and classification accuracy (See Methods for details). We selected the same number of top-ranked signatures from genes, isoforms, and combined profiles (combining gene and isoforms signatures together) to assess how useful these signatures would be for correctly separating patients with different stages. We used agglomerative hierarchical and k-means methods to implement consensus clustering. The results are similar (Figure 2). Overall, better performance was achieved with combined gene and isoform signatures than using gene and isoform signatures alone. The performance using isoform signatures deteriorated quickly with the increasing number of signatures. When the number of signatures increased from 140 to 220, for example, the classification stability score dropped from 0.52 to 0.47 and the number of misclassified patients increased from 57 (accuracy: 81.6%) to 63 (accuracy: 80.0%) using k-means based consensus clustering (Figure 2A). With hierarchical clustering, the classification stability score dropped from 0.49 to 0.43 and the number of misclassified patients increases from 54 (accuracy: 82.9%) to 75 (accuracy: 77.1%) (Figure 2B). In contrast, the performance using gene and combined signatures was more robust to the number of signatures used. These results suggest that isoform signatures are useful for separating cancer stages, but we should be careful about combining isoform information since more uninformative variables or noise would be introduced at such a high resolution level.

### Combining gene and isoform signatures provides biological meaningful results

**Figure 3:**
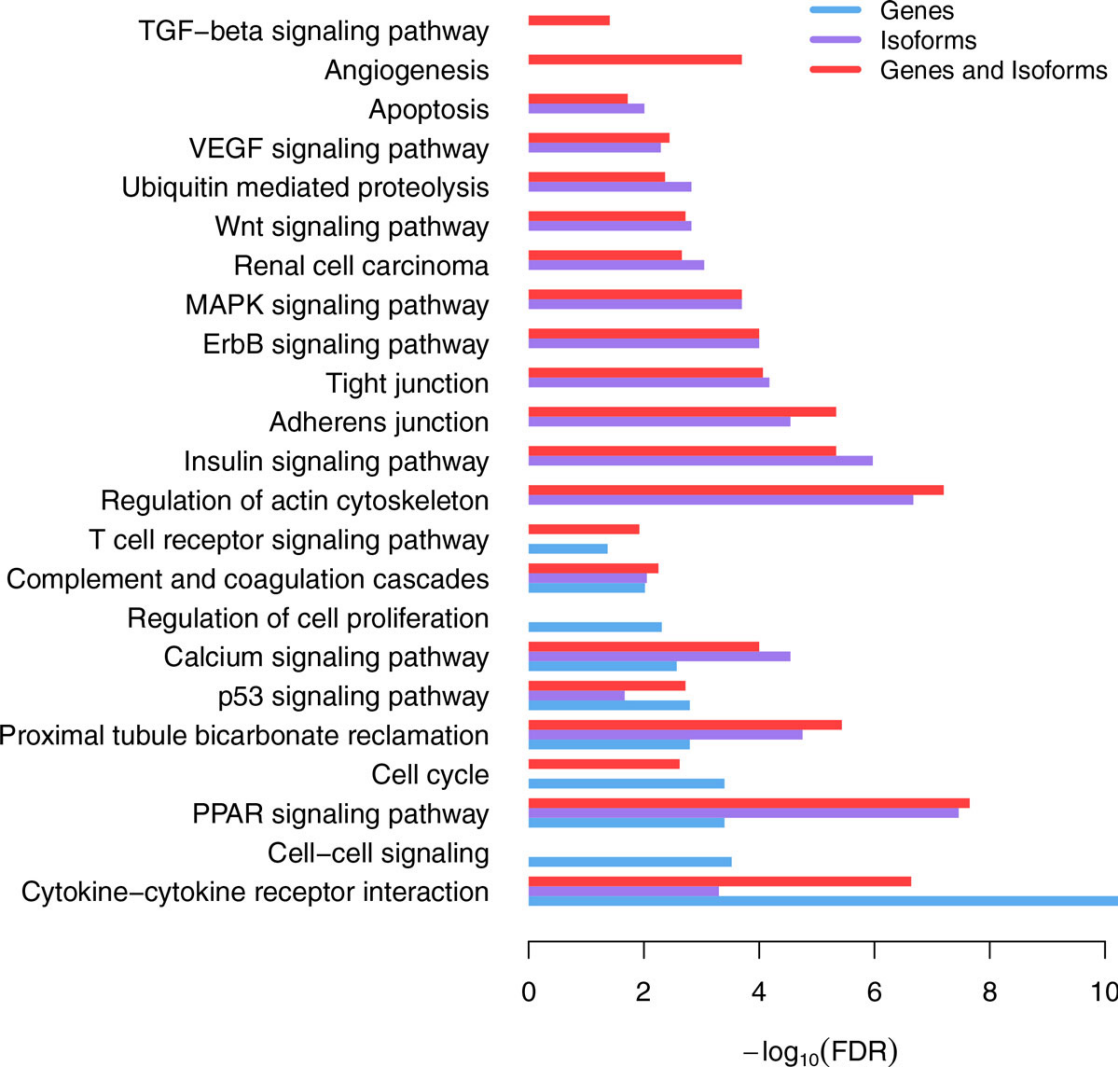
**Biological functions and pathways revealed by gene, isoform and combined signatures**.

Gene and isoform signatures associated with cancer stages were interpreted in GO biological process context as well as in KEGG pathway context (Figure 3, Additional File 5). A number of pathways involved in tumor growth, invasion, and metastasis were enriched in both gene and isoform signatures, which included cytokine-cytokine receptor interaction, PPAR signaling pathway, p53 signaling pathway, Calcium signaling pathway, etc. (Additional File 5). Cytokines and cytokine receptors are well known to be important contributors to cancer development and progression [39-42]. PPAR signaling is responsible for the regulation of cellular events that range from glucose and lipid homeostasis to cell differentiation and apoptosis, and there is emerging evidence indicating its anti-proliferative actions or tumor promoting effects [43]. Deregulation of calcium signaling is regarded as the primary event in the pathogenesis, growth, invasion, and secondary spread of cancer [44]. As an example, ITPKA was up-regulated in stage IV patients at both gene (log_2_FC = 2.14, FDR = 1.8e-10) and isoform levels (uc001znz.2, log_2_FC = 1.99, FDR = 1.1e-09). High expression of ITPKA has been reported to promote migration of tumor cells by two different mechanisms: ITPKA increases calcium entry that directly influences cell migration in EGF stimulated cells. In growth factor poor medium, ITPKA induces the formation of large cellular protrusions by stabilizing and bundling actin filaments [45].

In addition, there were important biological pathways uniquely identified by gene or isoform signatures. Cell cycle (FDR = 4.0e-04), cell-cell signaling (FDR = 3.0e-04), regulation of cell proliferation (FDR = 0.005), and T cell receptor signaling pathways (FDR = 0.05) were only observed by gene signatures, which are also known to be associated with tumor progression. For example, the overall mRNA of FOXA1 was highly expressed in stage IV patients (log_2_FC = 3.03, FDR = 2.6e-04). FOXA1 is involved in cell-cell signaling, and it promotes tumor progression in prostate cancer [46,47]. Adherens and tight junctions were only enriched in isoform signatures (FDR = 2.9e-06, FDR = 6.7e-05). Adherens junction is involved in establishing and maintaining cell-cell adhesion, and disruption of adherens junctions promotes tumor cell invasion and metastasis [48-50]. Tight junction is critical for maintaining cell to cell integrity and the loss of cohesion of the structure will lead to invasion and metastasis of cancer cells[51,52]. Besides, a number of signaling pathways well known to play a crucial role in cancer progression were only observed in isoform signatures, including ErbB signaling pathway, MAPK signaling pathway, Insulin signaling pathway, Wnt signaling pathway, VEGF signaling pathway, etc. These results suggest that isoform signatures provide additional insight into the biological mechanisms related to the tumor progression. The tight junction gene TJB2, for example, showed differential expression only at the isoform level (uc011lrs.2, log_2_FC=-2.46, FDR = 4.5e-06; uc011lrt.1, log_2_FC=-2.34, FDR = 1.8-04). TJP2 is a candidate tumor suppressor [53] and overexpression of TJP2 will block the cell cycle and inhibit cell proliferation [54].

Notably, combing gene and isoform signatures not only uncovered most of the biological processes detected by gene or isoform profiles but also suggested two additional critical pathways associated with cancer progression, angiogenesis (FDR = 2.0e-04) and TGF−beta signaling pathway(FDR = 0.04). Angiogenesis, the process of forming new blood vessels, allows cancer cells to make their own blood supply to obtain oxygen and nutrients, which leads to growth and metastasis [55-57]. The expression of 69 genes involved in angiogenesis was significantly changed at gene and/or isoform levels. 8 genes involved in the TGF-beta signaling pathway showed expression alternations at gene and/or isoform level (Additional File 5).

### Gene and isoform signatures predictive with clinical outcome

We used a Cox proportional hazard (PH) model to evaluate whether the detected gene and isoform expression signatures are predictive of the risk of cancer death. The 165 patients in stage II and stage III of KIRC were taken as an independent dataset and segregated into higher- and lower-than-median groups based on the expression level of the selected gene or isoform. Survival analysis was performed between these two groups. As a result, the expression level of 39 genes and 92 isoforms was found to be significantly associated with survival time (FDR ≤ 0.05). The 39 genes included ITPKA and RYR2 (calcium signaling), ITGA8 (regulation of actin cytoskeleton), FOXA1 and ACTN2 (cell-cell signaling), NPR3 (cell proliferation), etc. (Additional File 6). The 92 isoforms, corresponding to 86 genes, contained ITPKA (calcium signaling), ITGA8 (regulation of actin cytoskeleton), TJP2 (tight junction) and ACVR2A (cytokine-cytokine receptor interaction), AMOT and BAI1 (angiogenesis), etc. (Additional File 7). Most of these genes have been reported to be involved in cancer progress and metastasis in previous studies.

**Figure 4:**
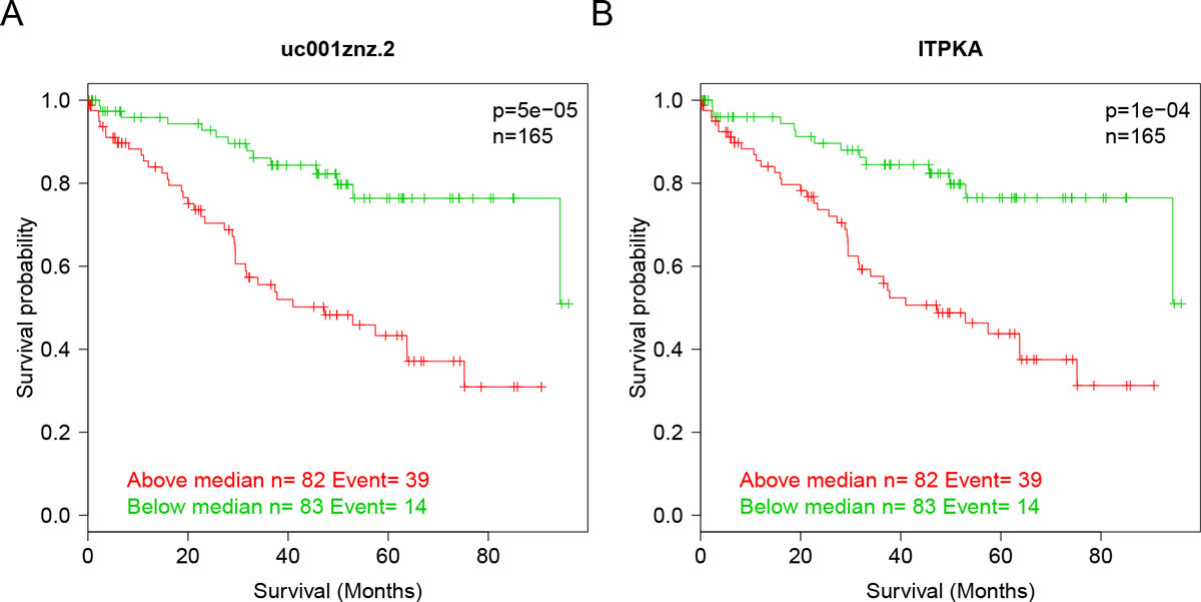
**The isoform (uc001znz.2) and gene expression of ITPKA are both predictive of survival in 165 stage II and III patients with KIRC**. Expression value which is lower than median is denoted in green and higher than median is noted in red. Patients with low expression value show a significantly higher median survival time compared with patients with high expression value (p = 5.0e-05 for the isoform uc001znz.2 and p = 1.0e-04 for ITPKA).

There were 8 genes whose overall mRNA and isoform expressions were both associated with clinical outcome, including ITPKA, ITGA8, OTOF, ZIC2, COL7A1, CILP, WDR72 and FLRT3. In these cases, the functional isoform dominated the gene expression, and thus a similar signal was obtained at both levels. Consistent with gene-level expression changes, for example, uc001znz.2, the major isoform of ITPKA (calcium signaling) was significantly up-regulated in the stage IV patients (log_2_FC = 1.99, FDR = 1.1e-09). In Kaplan-Meier estimates, patients with higher ITPKA expression in either isoform or gene level showed lower survival rates (Figures 4A and 4B). The median survival time was 94.3 months versus 47.2 months at both gene level and isoform level. In the univariate Cox PH model, the hazard ratios for ITPKA expression above median were 3.46 (p = 1e-04) at gene level and 3.67 (p = 5e-05) at isoform level. Multivariate Cox PH model analysis adjusting for age and gender was also performed, and ITPKA was also found to be significantly associated with survival time (p = 0.0005 at gene level and 0.0002 at isoform level). As we mentioned earlier, ITPKA is a cell motility-promoting protein that increases the metastatic potential of tumor cells. The expression of genes and isoforms associated with cancer stage and clinical outcome make ITPKA the potential target of advanced stage KIRC therapy.

**Figure 5:**
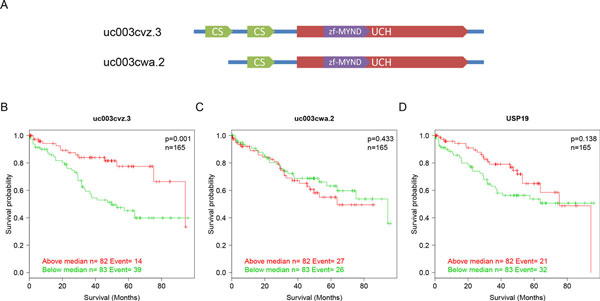
**Specific isoform expression of USP19 is predictive of survival in 165 stage II and III patients with KIRC**. Expression value which is lower than median is denoted in green and higher than median is noted in red. **(A) **Transcript structures of two isoforms, uc003cvz.3 and uc003cwa.2. **(B) **Patients with high expression value of uc003cvz.3 show a significantly higher median survival time compared with patients with low expression value (p = 0.001). **(C) **Patients with high expression value of uc003cwa.2 does not show a significantly higher median survival time compared with patients with low expression value (p = 0.433). **(D) **Patients with high expression value of the overall mRNA USP19 do not show a significantly higher median survival time compared with patients with low expression value (p = 0.138).

In some cases, however, background expression of nonfunctional isoforms added noise to gene abundance measurements and obscured the gene-level signal. Therefore, only the signal of functional isoforms could be observed. As an example, ubiquitin carboxyl-terminal hydrolase 19 (USP19), a deubiquitinating enzyme that regulates the degradation of various proteins and plays a role in cell proliferation and apoptosis, showed no significant difference on the overall mRNA expression between Stage I and Stage IV patients (log2 FC=-0.09, FDR = 0.12). Simultaneously, the overall mRNA expression of USP19 was not significantly associated with survival time (Figure 5D, p = 0.14). In contrast, uc003cvz.3, the major isoform of USP19 (the relative abundance ratio = 0.4), was significantly down-regulated in stage IV patients (log_2 _FC = -1.24 and FDR= 0.0003), and higher uc003cvz.3 expression suggested higher survival rates (Figure 5B). The median survival time for isoform uc003cvz.3 was 94.3 months versus 49.8 months. In the univariate Cox PH model, the hazard ratio for uc003cvz.3 expression above the median was 0.37 (p = 0.001). Multivariate Cox PH model analysis adjusting for age and gender was also performed and proved that the expression of isoform uc003cvz.3 was significantly associated with survival time (p = 0.0005). Besides the isoform uc003cvz.3, there was another isoform uc003cwa.2 expressed in similar abundance, which was not significantly changed between stages and was not associated with survival time (Figure 5C). Comparing the structure of these two isoforms, uc003cvz.3 and uc003cwa.2, we found uc003cvz.3 is longer at N terminal and more functionally important. Isoform uc003cwa.2 contains only one CS domain, while uc003cvz.3 has two CS domains (Figure 5A), which play an important role in the interaction of USP19 with the cellular inhibitor of apoptosis 2 and influence c-IAP1 and 2-dependent apoptosis [58]. These results suggest that the expression of the nonfunctional important isoform uc003cwa.2 obscures the changes of the overall mRNA expression level of UPS19 and that isoform-level analysis is sensitive to detect the signal of functional important isoforms.

## Discussion

Comparative analysis of expression alternations between early and late stage cancers improves our understanding of cancer growth and metastasis. Previous studies on gene expression profiles have identified overall mRNA expression changes in various kinds of cancers. These overall mRNA transcript level analyses, however, cannot uncover post-transcriptional deregulation and may underestimate the complexity of cancer progression. Recently, post-transcriptional deregulation such as splicing alternations, a key regulatory process by which functionally diverse isoforms can be expressed, has been reported to play an important role in cancer progression. The abundance of each individual isoform, which couples both transcriptional and post-transcriptional regulation, may serve as a valuable source to study the complexity of cancer progression.

RNA-seq technology, enabling a large dynamic range, high resolution, and low technical variance in measuring expression abundance, provides the opportunity of systematically comparing isoform expression profiles between early and late stage cancers. In this study, we not only identified stage-dependent gene and isoform expression signatures, but also compared the usefulness of these two kinds of signatures in terms of separating cancer stages, biological relevance, and prediction of clinical outcome. Remarkably, about 80% of genes with significant isoform expression changes do not exhibit alternations at the overall mRNA level. These isoforms are useful for separating cancer stages and are enriched in a number of critical biological function and pathways associated with cancer progression and metastasis, such as adherens and tight junctions, ErbB signaling, MAPK signaling, VEGF signaling pathways, etc. Furthermore, the expression abundance of a number of isoforms is significantly associated with the increased risk of death in an independent dataset. These results demonstrate that isoform expression profiling provides unique and important information that cannot be detected by the gene-level. Isoform-level analysis complements the gene-level analysis, and combining gene and isoform signatures improves the classification performance and presents a comprehensive view on the potential biological mechanisms involved in cancer progression.

Moreover, differential expression observed at the isoform level but not at the gene level provides an opportunity for exploring potential post-transcriptional regulatory mechanisms to gain insights into isoform specific regulation. Among 1637 genes with isoform expression changes, only 17 genes contain two or more isoforms showing opposite expression changes, which suggests that isoform switching is not likely to be a major contributor to splicing pattern changes in cancer progression. To find RNA binding proteins responsible for modulating splicing during cancer progression, we can identify stage-dependent splicing pattern changes based on the ratio of alternative spliced isoforms and search for overrepresented nucleotide sequences near stage-associated splicing events. Additionally, analyzing the 3' UTR of genes with differentially expressed isoforms is one way to find the miRNA involved in cancer progression.

Although profiling of individual isoforms provides useful information, we should be careful when we interpret the results from such a high resolution level. Read assignment uncertainty inherent in the RNA-seq data analysis may introduce noise and false positives. Some reads cannot be assigned unequivocally to an isoform since many isoforms share exons. This read assignment uncertainty will affect the accuracy of isoform expression quantification and introduce noise, especially for low abundance genes with multiple isoforms. This is possibly the reason why classification performance drops quickly with the increasing number of isoform expression signatures. On the other hand, many isoforms could be non-functional noise. As a result, the isoforms detected may simply reflect noisy splicing and are not likely to be translated into functional proteins [59,60]. For example, one isoform of MLH3 (uc010tuy.1), a DNA mismatch repair gene without significant changes at the overall mRNA level (log_2_FC=-0.04, FDR = 0.6), was significantly downregulated in the late stage of cancer (log_2_FC=-1.71, FDR = 0.006). However, this isoform is vulnerable to nonsense-mediated decay and cannot be translated into protein. As another example, one isoform of MGRN1 (uc010uxq.1) with significant expression changes (log_2_FC = 2.53, FDR = 0.001) was also a non-coding transcript. Consistently, a previous study has reported increased levels of noisy splicing in cancers, leading to marked changes in premature stop-codon frequency for tumor suppressor and oncogenes[60]. Thus it is important to consider splicing noise when identifying stage-dependent isoform expression signatures. To reduce the effect of noisy splicing and read assignment uncertainty, summarizing the reads into more functional important units, e.g., including only reads that map to the coding sequences may be more suited for finding biologically meaningful results.

## Conclusion

Isoform expression profiling extends our knowledge about cancer progression and serves as a useful complement to gene level analysis. Combining gene and isoform expression signatures helps to identify advanced stage cancers and present a comprehensive view on biological mechanisms in cancer development and progression.

## Competing interests

The authors declare that they have no competing interests.

## Authors' contributions

YS led the project and oversaw the analysis. QL and SLZ designed and performed the research. PFS participated in the survival analysis. QL and SLZ wrote the manuscript. All authors have read and approved of the final manuscript.

## Supplementary Material

Additional file 1 **The distribution of the relative abundance ratio of "major" isoform to the corresponding gene**. (A) The ratio distribution for all genes. (B) The ratio distribution for genes with two or more isoforms expressed. Click here for file

Additional file 2 **Differentially expressed genes between the early and the late stages of KIRC (FC≥2 & FDR≤0.001)**. Click here for file

Additional file 3 **Differentially expressed isoforms between the early and the late stages of KIRC (FC≥2 & FDR≤0.001)**. Click here for file

Additional file 4 **17 genes have two or more isoforms differentially expressed with opposite change directions**. Click here for file

Additional file 5 **Functional enrichment results in gene, isoform and combined signatures**. Click here for file

Additional file 6 **39 genes whose expression abundance is predictive of increased risk of cancer death in stage II and III patients**. Click here for file

Additional file 7 **92 isoforms whose expression abundance is predictive of increased risk of cancer death in stage II and III patients**. Click here for file

## Declarations

The publication costs for this article were funded by the corresponding author.

This article has been published as part of *BMC Systems Biology *Volume 7 Supplement 5, 2013: Selected articles from the International Conference on Intelligent Biology and Medicine (ICIBM 2013): Systems Biology. The full contents of the supplement are available online at http://www.biomedcentral.com/bmcsystbiol/supplements/7/S5.
