# Supplementary figures and images for: Gene and isoform expression signatures associated with tumor stage in kidney renal clear cell carcinoma
Source: BMC Syst Biol. 2013 Dec 9;7(Suppl 5):S7. doi: 10.1186/1752-0509-7-S5-S7 (PMC4028983; doi:10.1186/1752-0509-7-S5-S7)

A

All Genes

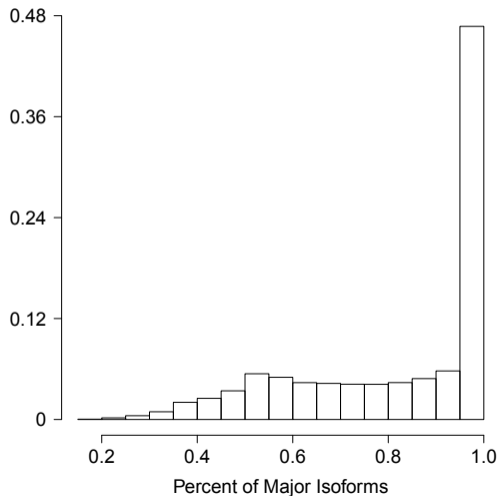

B

Genes with more than two Isoforms

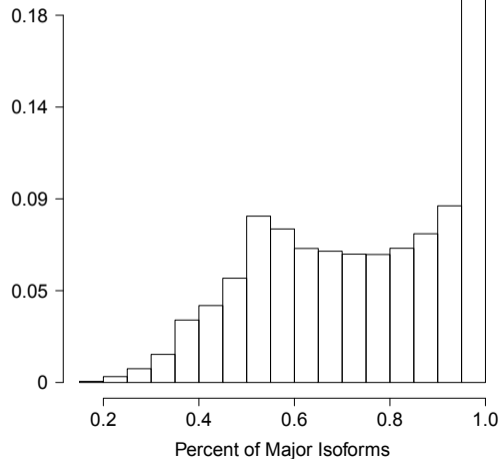

Supplement: Additional file 1 — The distribution of the relative abundance ratio of "major" isoform to the corresponding gene. (A) The ratio distribution for all genes. (B) The ratio distribution for genes with two or more isoforms expressed. [file 1752-0509-7-S5-S7-S1.PDF]
